# Variation in the Frequency and Extent of Hybridization between *Leucosceptrum japonicum* and *L*. *stellipilum* (Lamiaceae) in the Central Japanese Mainland

**DOI:** 10.1371/journal.pone.0116411

**Published:** 2015-03-04

**Authors:** Yue Li, Masayuki Maki

**Affiliations:** 1 Division of Plant Evolutionary Biology, Department of Environmental Life Sciences, Graduate School of Life Sciences, Tohoku University, Aoba, Sendai 980–8578, Japan; 2 Botanical Gardens, Tohoku University, Kawauchi 12–2, Aoba, Sendai 980–0862, Japan; United States Department of Agriculture, UNITED STATES

## Abstract

Variations in the frequency and extent of hybridization among mixed populations located in the same contact zone provide natural laboratories for the study of extrinsic reproductive isolation maintaining species integrity. In this study, we examined the pattern of hybridization between *L*. *japonicum* and *L*. *stellipilum* among mixed populations in different localities of a contact zone. The genetic structures from three sympatric populations and six mixed populations in the hybrid zone, and five reference populations far from the contact zone, were characterized using 10 neutral nuclear microsatellite markers. Evidence from genetic distance-based clustering analysis, the frequency distribution of admixture proportion values, and the hybrid category assignment approaches indicated that the frequency and extent of hybridization varied considerably among populations in the contact zone between *L*. *japonicum* and *L*. *stellipilum*. One likely explanation is that variation in exogenous (ecological) selection among populations might contribute to differences in frequency and extent of hybridization. The present study will facilitate future research exploring the evolution of reproductive isolation between *L*. *japonicum* and *L*. *stellipilum*.

## Introduction

Natural hybridization is most often the result of secondary contact by range expansion between previously isolated populations or may arise via primary intergradation along an environmental gradient during the process of parapatric speciation [1]. Natural hybridization is a widespread phenomenon in plant species and occurs in 40% of vascular plant families [2–3]. The frequency of natural hybridization in plants varies among families, genera, and species pairs [4].

The frequency of hybrid formation between species pairs can reflect the strength of reproductive isolation barriers between species and the stages in their speciation process [5–6]. Hybridization frequency varies at the species level because the period required for the development of reproductive isolation in the speciation process is different between species pairs that experience unique evolutionary histories [7–8]. When the frequency of hybridization between parental types is relatively low, contact sites are predominated by the parental or backcross type with few intermediate types (bimodal). The bimodal contact zone is strongly associated with well-developed (but incomplete) pre-zygotic isolation, suggesting that speciation of parental forms is nearly complete [6]. In contrast, when the frequency of hybridization between parental types is sufficiently high, contact sites consist mainly of the intermediate type (unimodal), indicating that pre-zygotic isolation is largely incomplete [6]. The frequency of hybridization can vary among hybridizing species pairs: some are composed largely of F_1_ individuals (*Phlox*: [9]; *Phyllodoce*: [10–11]; *Typha*: [12]; *Rhizophora*: [13]; *Rhododendron*: [14–15]; *Anacamptis*: [16]; *Quercus*: [17]), a small proportion of F_1_ with many backcrosses (*Populus*: [18]; *Arctostaphylos*: [17]; *Helianthus*: [19]), mainly advanced generation backcrosses (*Liparis*: [20]; *Iris*: [21]; *Silene*: [22–23]; *Vasconcellea*: [24]), and mainly parental types with a few hybrids (*Oenanthe*: [25]; *Lasthenia*: [26]).

However, variations in contact zone modality were observed not only between species pairs, but also across the different contact sites of a single species pair (*Phlomis* [27–28], *Ipomopsis* [29–30], *Senecio* [31], *Epimedium* [32], *Dactylorhiza* [33], *Pinus* [34], *Quercus* [35–39]). These observations suggest that the strength of reproductive isolation between a species pair is not similar everywhere they come into contact but can be affected by local conditions. However, this variation can also be influenced by evolutionary and demographic history; e.g., an old contact zone will be characterized by bimodality and a relatively recent contact zone will be characterized by unimodality [40]. The influences of the evolutionary and demographic history differences among contact sites can be eliminated only by studying some mixed populations or contact sites located in the same contact zone because the contact times in these populations are not expected to be largely different from each other.

Contact zones can provide natural laboratories for the study of reproductive isolation between related species [2, 41–42]. Estimating the factors involved in the initial limitation of gene flow and that drive speciation by examining present day reproductive isolation is difficult because current reproductive isolation also includes some isolation mechanisms that evolved after species divergence. Contact sites with various levels of isolation breakdown may represent different levels of differentiation between species pairs along the gradual speciation continuum and will therefore affect the production of hybrids. Differences in the strength of reproductive isolation can be expressed as serial combinations of sequentially acting pre-zygotic (dispersal ability, flowering phenology, pollination system, pollen performance) and post-zygotic (fruit abortion, seed inviability, hybrid inviability, hybrid sterility) reproductive isolation [43–44]. In the absence of an intrinsic reproductive isolation barrier among contact sites, examination of the differences in isolation barriers between unimodal contact sites (with weaker isolation barriers) and bimodal contact sites (with stronger isolation barriers) could help to pinpoint extrinsic reproductive isolation mechanisms limiting gene flow between species and thus promote speciation [45].

Geographic variation in pre-zygotic isolation, such as dispersal ability, pollinator preference, and pollen performance, can be affected by several factors, including vegetation type [34], floral phenotype [32, 46], temperature [46], and edaphic conditions [35, 47]. Geographic variation in post-zygotic reproductive isolations is mainly affected by the abiotic environment (genotype × environment interactions), although the first step of hybrid breakdown is generally genomic incompatibility [48]. Variation in fine-scale habitats within a site would promote differences in the strengths of selection on certain genotypes and lead to variable fitness and high levels of spatial structure of certain genotypes within the site [21, 49–50]. Fine-scale habitat may differ among localities and will lead to differences in the variation of population structure among contact sites. For example, the absence of suitable habitats for intermediate genotypes would reduce their survivorship [26, 51–52]. In contrast, a hybrid zone dominated by F_1_ is explained by habitat-mediated superiority of F_1_s over all other genotype classes compared to other sites [14–15]. Quantification of the fine-scale environmental differences between sites within a contact zone may provide insight into specific environmental variables associated with reproductive isolation [23, 49–53]. Therefore, to better understand the evolution of reproductive isolation along speciation process, the first step is to characterize variation in hybridization frequency within a hybrid zone of a single species pair.

The frequency of hybridization may not be accessible when contact zone structures are examined by morphological approaches. Hybrids are not always phenotypically intermediate between pure species, but often display a combination of intermediate characteristics and parental and extreme characteristics [54]. Moreover, the frequency of hybridization may be different among neutral phenotypic traits and those under divergent selection [55]. The latter traits will be less prone to introgress across species boundaries than neutral traits [56–57]. The choice of traits to be examined will affect estimates of hybridization frequency. For example, because phenotype distribution may be strongly bimodal despite ongoing extensive interspecific gene exchange [58], morphological investigation alone is insufficient to discriminate introgressants from pure individuals [59–61]. In such situations, neutral markers will be useful for examining the frequency of hybridization because they are prone to introgression across species boundaries.

The genus *Leucosceptrum* Smith consists of suffrutescent herbs or subshrub taxa, which are endemic to East Asia. This genus is composed of five species. Of these, *Leucosceptrum japonicum* and *Leucosceptrum stellipilum* are endemic to Japan [62]. *Leucosceptrum japonicum* is distributed mainly in the eastern part of the Japanese mainland and is sporadically distributed in the western part, while *L*. *stellipilum* is restricted to the western part of the Japanese mainland. *Leucosceptrum japonicum* often grows at an elevation of 50–2000 m, while *L*. *stellipilum* grows at 30–1000 m. Both species flower in early autumn, although *L*. *stellipilum* tends to flower later than *L*. *japonicum* in the same local area. Bumblebees are common pollinators of the two species. *Leucosceptrum japonicum* and *L*. *stellipilum* are different in several morphological characters: the leaf blades of *L*. *japonicum* are oblong or widely lanceolate and sparsely pilose or glabrous, while those of *L*. *stellipilum* are widely elliptic or elliptic—obovate and densely stellate pubescent. Flowers of *L*. *japonicum* are pale yellow, while those of *L*. *stellipilum* are rose purple.

The two species occur in the same population in which the geographic distribution of *L*. *japonicum* and *L*. *stellipilum* overlap on the central Japanese mainland. Hybridization between *L*. *japonicum* and *L*. *stellipilum* was initially documented based on vegetative and floral characters [63]. In the central part of the Japanese mainland where the distributions of *L*. *japonicum* and *L*. *stellipilum* overlap, hybridization occurring in several contact sites has been described based on morphological characters and species-specific genetic markers of cpDNA spacer and nrITS [64]. However, analysis of only one nuclear region (nrITS) cannot provide sufficient information to delineate genealogical classes (e.g., parental type, first generation hybrids, second generation hybrids, and first generation backcrosses to either parental-type direction). Thus, later generation hybrids and introgressants may also have been underestimated by the previous study. It is necessary to examine the frequency of hybridization in each locality by other suitable neutral molecular markers.

To gain a better understanding of whether the frequency of hybridization between *L*. *japonicum* and *L*. *stellipilum* can be influenced by local conditions, we examined the patterns of hybridization between the two species among mixed populations in different localities of a hybrid zone. We performed a population genetic study based on 10 microsatellite markers, and examined the degree of variation in frequency and extent of hybridization between *L*. *japonicum* and *L*. *stellipilum* among mixed populations.

## Materials and Methods

### Ethics Statement

No specific permissions were required for the field studies of all the sampling locations. The sites for our samplings did not belong to the protect areas or private lands. The field studies did not involve endangered or protected species. The GPS coordinates of our study were showed in Table 1 of our manuscript.

**Table 1:** Population code, collection locality, geographical coordinates and number of individuals (*N*) per population studied with morphology and microsatellite loci.

Population code	Locality	Latitude (N)	Longitude (E)	Altitude (m)	Type inferred from morphology and location	*N*
J1	Sakunami	38°19′ 12″	140°34′ 48″	739	Allopatric *L*. *japonicum*	24
J2	Oguni	38°3′ 36″	139°51′ 36″	371	Allopatric *L*. *japonicum*	20
S1	Kumano-magosetouge	34°6′ 0″	136°12′ 36″	43	Allopatric *L*. *stellipium*	18
S2	Kumano-okubo	33°58′ 12″	136°6′ 36″	401	Allopatric *L*. *stellipium*	13
S3	Kumano-tamaokiguchi	33°53′ 38″	135°52′ 12″	200	Allopatric *L*. *stellipium*	12
H1	Kunimi	35°28′ 12″	136°25′ 12″	753	Sympatric *L*. *japonicum*	13
H2	Shinatamatouge	35°31′ 48″	136°24′ 36″	876	Sympatric *L*. *japonicum*	15
H3	Minami-machi	35°40′ 12″	136°54′ 36″	319	Sympatric *L*. *stellipium*	15
H4	Horado	35°30′ 0″	136°49′ 12″	185	Mixed population	20
H5	Kami-ishidu	35°15′ 0″	136°25′ 12″	395	Mixed population	27
H6	Kanzaki	35°39′ 36″	136°42′ 36″	387	Mixed population	56
H7	Kuze	35°33′ 36″	136°31′ 12″	296	Mixed population	22
H8	Noharatani	35°27′ 36″	136°29′ 24″	317	Mixed population	32
H9	Kozanshi	36°7′ 41″	137°11′ 10″	644	Mixed population	26

### Study sites and sampling

**Fig 1:**
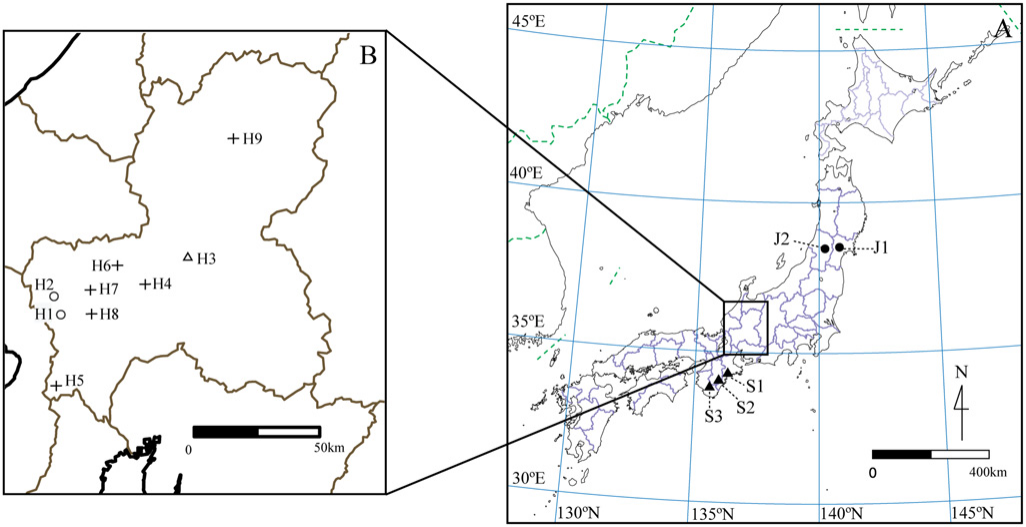
Hybrid zone between *L*. *japonicum* and *L*. *stellipilum* in the central Japanese mainland. (A) Map of Japan showing locations of the hybrid zone and allopatric pure *L*. *japonicum* populations (filled circles) and pure *L*. *stellipilum* (filled triangles) populations. (B) Detailed map of the hybrid zone illustrating the sampling sites of sympatric *L*. *japonicum* (open circles), sympatric *L*. *stellipilum* (open triangle), and putative hybrids (cross). Additional details for each population are shown in Table 1.

*Leucosceptrum japonicum* is distributed mainly in the eastern part of the Japanese mainland and is sporadically distributed in the western part, while *L*. *stellipilum* is restricted to the western part of the Japanese mainland. The distributions of these species overlap along the central part of the Japanese mainland. Six mixed populations of *L*. *japonicum* and *L*. *stellipilum* (H4–H9), two sympatric populations of *L*. *japonicum* (H1 and H2), and one sympatric population of *L*. *stellipilum* (H3) located in this region were chosen as study populations (Fig. 1, Table 1). Previously, multivariate analyses of morphological characters showed that the mixed populations contained both *L*. *japonicum* and *L*. *stellipilum*, as well as morphologically intermediate plants [64]. In addition, two allopatric populations (populations distant from the hybrid zone: J1 and J2) of *L*. *japonicum* and three allopatric populations (S1, S2, and S3) of *L*. *stellipilum*, which were unaffected by interspecific gene flow, were sampled as references. Twelve to 57 individuals were randomly collected at each site. Leaf samples were collected from individuals at least a few meters apart to reduce the chance of resampling the same individual. Leaf tissues were stored at—70°C until DNA extraction.

### Microsatellite amplification and screening

Genomic DNA was extracted from 50–100-mg samples of freeze-dried leaf tissues with DNeasy Plant Mini Kits (Qiagen, Hilden, Germany), the cetyl trimethyl ammonium bromide (CTAB) method, or a modified CTAB method [65]. DNA concentrations and purities were measured using a NanoDrop ND-1000 Spectrophotometer (Thermo Fisher Scientific, Waltham, MA). All individuals sampled from the 14 populations were genotyped for 10 simple sequence repeat (SSR) loci developed for *L*. *japonicum* and *L*. *stellipilum* by Li and Maki [66]: Leu1, Leu2, Leu3, Leu4, Leu5, Leu6, Leu7, Leu8, Leu9, and Leu10. Amplification and data analyses were performed according to the procedures described by Li and Maki [66].

### Microsatellite diversity and population genetic analyses

Genetic diversity was measured as the mean number of alleles per locus (*N*_a_); observed (*H*_O_), expected (*H*_E_), and heterozygosities were calculated using the program GenAlEx version 6 [67]. The overall levels of genetic differentiation of *L*. *japonicum* populations and *L*. *stellipilum* populations were assessed by calculating pairwise *F*_ST_ values with 1000 permutations in Arlequin version 3.5 [68]. Weir and Cockerham’s [69] estimate of the inbreeding coefficient (*F*_IS_) for each population was determined using the program GenAlEx version 6 [67]. Heterozygote deficits are commonly applied to quantify bimodal populations [6]. Positive, negative, and zero values of *F*_IS_ indicate heterozygote deficit, heterozygote excess, and Hardy—Weinberg equilibrium (random mating), respectively. As a measure of genetic relatedness between individuals, Nei’s pairwise genetic distance (*D*_A_) [70] was calculated using POPULATIONS v.1.2.30 [71]. Genetic grouping of the allopatric, sympatric and mixed populations was visualized by principal coordinate analysis (PCO) based on *D*_A_ using R [72].

### Population structure and hybridization assignment

A Bayesian assignment approach implemented in the program STRUCTURE version 2.2.3 [73] was used to estimate the proportion of each individual’s genome originating from each of the parental populations. Different probable values of *K* were estimated for all samples under the admixture model with independent allele frequencies, and 10 replicate runs were performed for each value of *K* ranging from 1 to 10 with a burn-in of 50,000 steps followed by 100,000 Markov chain Monte Carlo (MCMC) simulations. The optimal value of *K* was calculated using the method of Pritchard et al. [73] and Evanno et al. [74] and the output was interpreted by post-processing all runs using STRUCTURE HARVESTER [75]. The results from the 10 replicates were averaged using CLUMPP [76] and the output was displayed using DISTRUCT 1.1 [77]. STRUCTURE calculated the admixture proportion (*q*) for each individual originating from the *L*. *japonicum* cluster (*q*_*1*_) and the *L*. *stellipilum* cluster (*q*_*2*_). The admixture proportion of the *L*. *japonicum* cluster (*q*_*1*_) was used to assign individuals into three categories. Individuals with a *q*_*1*_ value ranging between 0.9 and 1.0 were treated as *L*. *japonicum*, while those with values from 0 to 0.1 were treated as *L*. *stellipilum* and those from 0.1 to 0.9 were treated as hybrids. Furthermore, we assessed the modality of each population by testing the distribution of admixture proportions (*q*_*1*_-value) calculated in STRUCTURE using Hartigan’s dip test. This test measures multimodality as the maximum difference over all sample points between the empirical distribution, and the unimodal distribution minimizes the maximum difference [78]. The dip test rejects the null hypothesis of unimodality at a significance level of 0.05. Dip statistics were attained using the “diptest” package [79] in R [72].

A model-based Bayesian approach implemented in NEWHYBRIDS version 1.1 [80] was used to calculate the posterior probability that an individual falls into one of six different categories—each pure parental type (P1 and P2, respectively), first generation hybrids (F_1_), second generation hybrids (F_2_), and first generation backcrosses to either parental-type direction (BC-P1 and BC-P2, respectively). NEWHYBRIDS was run independently five times with no prior information using the Jeffreys-like priors and 200,000 MCMC sweeps after 50,000 burn-in steps. A threshold value (*Tq*) of 0.5 was used to indicate that the hybrids were correctly assigned to their respective categories. If an individual could not be clearly assigned to a specific genotypic class (posterior probability < 0.5 in the assignment of any genotype class) using NEWHYBRIDS, although it was considered of hybrid origin (assignment probability 0.1–0.9) using STRUCTURE, such individuals were considered beyond second generation hybrids, which NEWHYBRIDS does not normally attempt to identify [81]. In such cases, following the approach of Field *et al*. [82], classes were broadly defined as later generation classes (generation unknown); i.e., *q*_*1*_-values of 0.7–0.9, 0.1–0.3, and 0.3–0.7 indicated backcross to *L*. *japonicum*, backcross to *L*. *stellipilum*, and advanced generation hybrid (F_n_), respectively. Note that the Bayesian-based assignment approach may assign later generation backcrosses to parental categories due to insufficient numbers of loci [83]. However, misdiscrimination of later generation backcrosses to parental categories is not problematic because later generation backcrosses will show similar performance to pure individuals [21,50]. The modality of the contact zone depends on proportions of the parental genotype and intermediate genotype. F_1_, F_2_, and F_n_ were treated as intermediate genotypes, while both backcross categories (early generation and later generation) and parental categories were treated as parental genotypes.

### Genotype simulation and assignment

To evaluate the power of microsatellites for detecting and delineating hybrids, we performed assignment tests on a data set containing four groups of simulated hybrid genotypes (F_1_, F_2_, and first generation backcrosses with each parental species) and two groups of parental genotypes (*L*. *japonicum* and *L*. *stellipilum*). To obtain pure parental genotypes to generate simulated genotypes, initial runs of STRUCTURE were performed to detect potential introgressants. Genotypes that had an admixture proportion (*q*) > 0.90 in their respective clusters were used to generate simulated genotypes. Forty-four *L*. *japonicum* and 43 *L*. *stellipilum* individuals from allopatric populations were used to obtain pure parental genotypes. Fifty simulated genotypes from each of the parental and hybrid classes were then obtained using HYBRIDLAB 1.0 [84]. The efficiency (number of individuals correctly assigned), accuracy (proportion of an identified group that truly belongs to that category), and performance (efficiency multiplied by accuracy) of this Bayesian analysis were evaluated according to the method of Vähä and Primmer [85]. The simulated genotypes were also analyzed using NEWHYBRIDS with no prior information using the Jeffreys-like priors and 200,000 MCMC sweeps after 500,00 burn-in steps. A threshold value of 0.5 was used to indicate that the hybrids were correctly assigned to their respective categories in NEWHYBRIDS.

## Results

### Microsatellite diversity

The mean number of alleles per locus (*N*_a_) within the population varied from 4.50 to 10.50 (Table 2). The *H*_O_ ranged from 0.443 to 0.709 (Table 2), and the *H*_E_ ranged from 0.508 to 0.750 (Table 2). All pairwise *F*_ST_ estimates were significantly different from zero (*P* < 0.05).

**Table 2:** Mean number of alleles per locus (*N*_a_), observed heterozygosities (*H*_O_), expected heterozygosities (*H*_E_)and fixation index (*F*_IS_) estimated at ten microsatellite loci in *L*. *japonicum*, *L*. *stellipium* and their hybrids.

Population code	*N*_a_	*H*_O_	*H*_E_	*F*_IS_
J1	5.300 (0.978)	0.517 (0.079)	0.553 (0.077)	0.073 (0.032)
J2	6.000 (0.894)	0.629 (0.079)	0.645 (0.077)	0.029 (0.028)
S1	5.600 (0.991)	0.531 (0.095)	0.587 (0.106)	0.084 (0.036)
S2	4.500 (0.860)	0.509 (0.108)	0.550 (0.101)	0.110 (0.085)
S3	5.400 (1.035)	0.541 (0.097)	0.560 (0.099)	0.032 (0.025)
H1	5.600 (0.897)	0.569 (0.084)	0.618 (0.072)	0.124 (0.087)
H2	5.400 (0.777)	0.573 (0.076)	0.644 (0.060)	0.142 (0.078)
H3	5.100 (1.090)	0.473 (0.097)	0.508 (0.098)	0.067 (0.049)
H4	7.400 (1.249)	0.485 (0.081)	0.655 (0.063)	0.311 (0.093)
H5	8.100 (1.269)	0.443 (0.079)	0.703 (0.047)	0.396 (0.087)
H6	10.500 (1.249)	0.516 (0.051)	0.750 (0.034)	0.318 (0.047)
H7	6.100 (0.781)	0.564 (0.092)	0.697 (0.039)	0.205 (0.102)
H8	9.000 (1.065)	0.507 (0.084)	0.736 (0.045)	0.338 (0.087)
H9	4.800 (0.467)	0.709 (0.064)	0.594 (0.043)	-0.199 (0.078)

The overall genetic differentiation was considerable in the comparison between *L*. *japonicum* populations and *L*. *stellipilum* populations with *F*_ST_ ranging from 0.29 to 0.37 (S1 Table). In contrast, lower levels of genetic differentiation were detected between populations within *L*. *japonicum* (*F*_ST_ range: 0.16–0.25) and *L*. *stellipilum* (*F*_ST_ range: 0.05–0.14) (S1 Table). Fixation indices (*F*_IS_s) were not significantly different from zero (random mating) at all loci for the allopatric *L*. *japonicum* and *L*. *stellipilum* populations (S2 Table). In contrast, significant heterozygote deficits (positive *F*_IS_ value) were detected at some loci in populations H4–H8, while significant heterozygote excess (negative *F*_IS_ value) was observed at some loci in population H9 (S2 Table). All loci showed polymorphisms, except loci Leu4, Leu7, and Leu8, which have fixed alleles in some populations (S2 Table).

### Genetic distance-based clustering analysis

**Fig 2:**
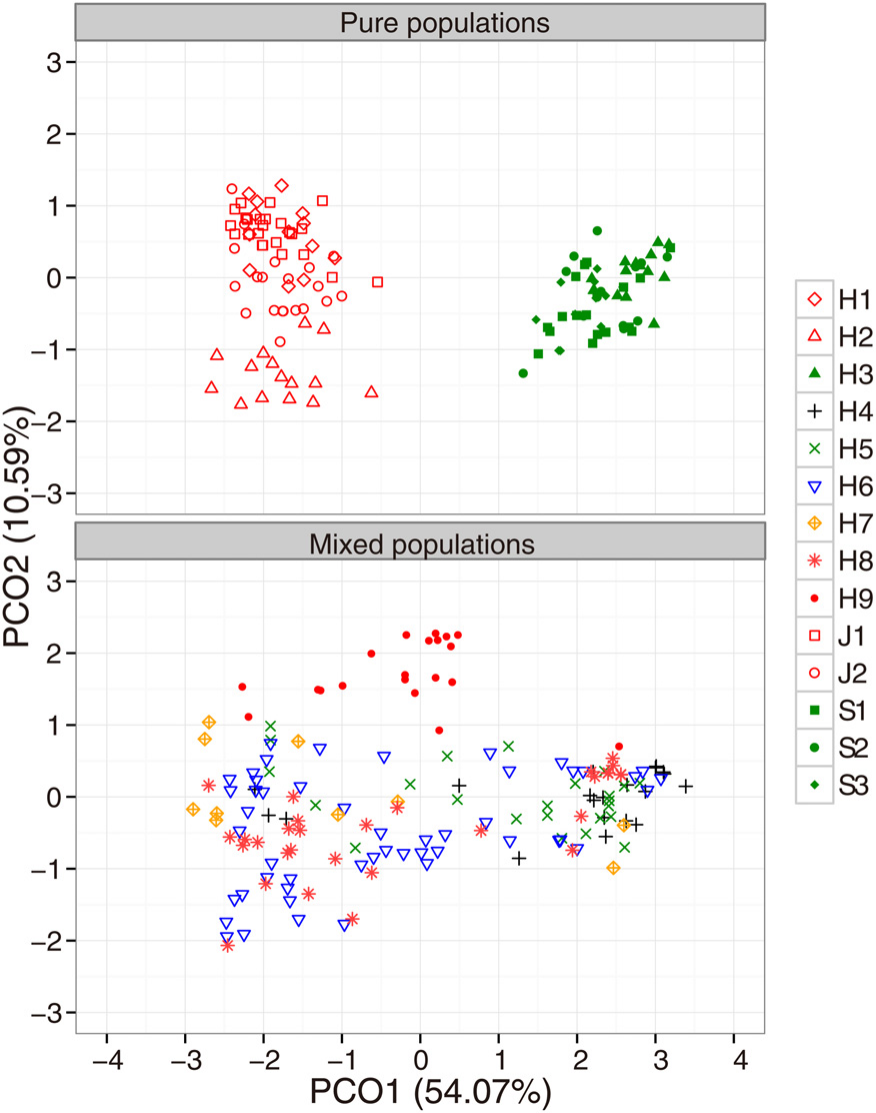
Principal coordinate analysis of pairwise Nei’s distance at 10 microsatellite loci in morphological pure populations (J1, J2, S1, S2, S3, H1, H2, and H3) and mixed populations (H4–H9).

Fig. 2 shows the PCO plots of each population based on individual genetic distances. The first and second axes accounted for 58.91% and 9.32%, respectively, of the total variation in PCO on microsatellite genotypes (Fig. 2). Two isolated groups were found to correspond to *L*. *japonicum* and *L*. *stellipilum* along the first axis. Sympatric *L*. *japonicum* (H1 and H2) and *L*. *stellipilum* (H3) populations closely clustered within allopatric *L*. *japonicum* and *L*. *stellipilum* pure sites (Fig. 2). Other individuals from the contact zone including populations H4–H9 covered the whole variation range of both species as well as intermediate positions. However, the distribution range differed among populations. In population H9, abundant individuals were distributed in the intermediate position, while only a few individuals were clustered within *L*. *japonicum* or *L*. *stellipilum*. In contrast, in populations H4 and H7, few individuals were distributed in the intermediate position, while most individuals were clustered within *L*. *japonicum* and *L*. *stellipilum*. Most individuals were clustered within *L*. *stellipilum* in H4, while most individuals were clustered within *L*. *japonicum* in H7. In populations H5, H6, and H8, a few individuals were distributed in intermediate positions, while others were clustered within *L*. *japonicum* and *L*. *stellipilum*.

### Genetic admixture analysis

**Fig 3:**
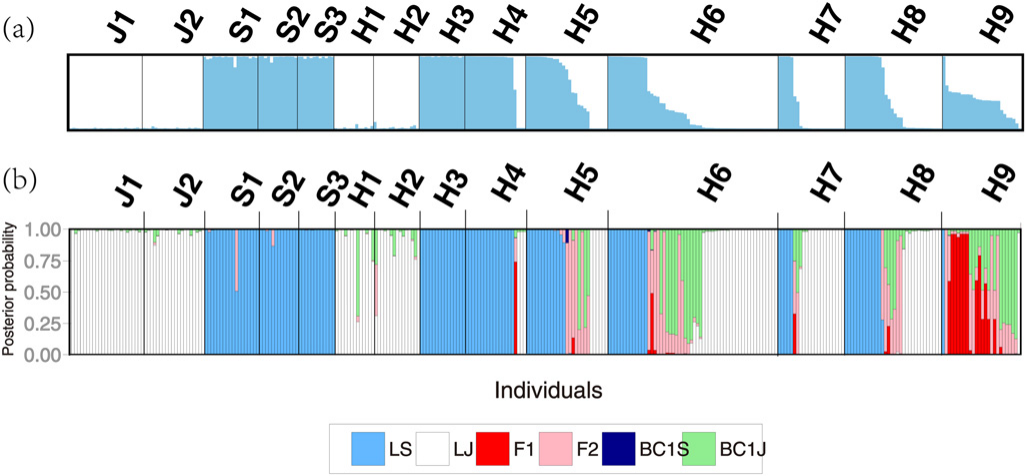
Genetic variation in pure *L*. *japonicum* populations, pure *L*. *stellipilum* populations, sympatric *L*. *japonicum*, sympatric *L*. *stellipilum*, and putative hybrids. (a) Admixture analyses showing the proportion of the genome of each individual originating from *L*. *japonicum* or *L*. *stellipilum* using the program STRUCTURE. Each individual is represented as a vertical bar divided into two segments representing the proportion of the genome from each of the genetic groups of *L*. *japonicum* (white) or *L*. *stellipilum* (blue). (b) Posterior probabilities of the genotype class estimated with NEWHYBRIDS. Each individual is represented as a vertical bar divided into six segments. Each color indicates the posterior probabilities of an individual assignment to pure *L*. *japonicum* (LJ), pure *L*. *stellipilum* (LS), F_1_, F_2_, and first generation backcross of a F_1_ hybrid with a pure *L*. *japonicum* (BC1J) or with a pure *L*. *stellipilum* (BC1S). Populations are labeled above the bar plots.

**Fig 4:**
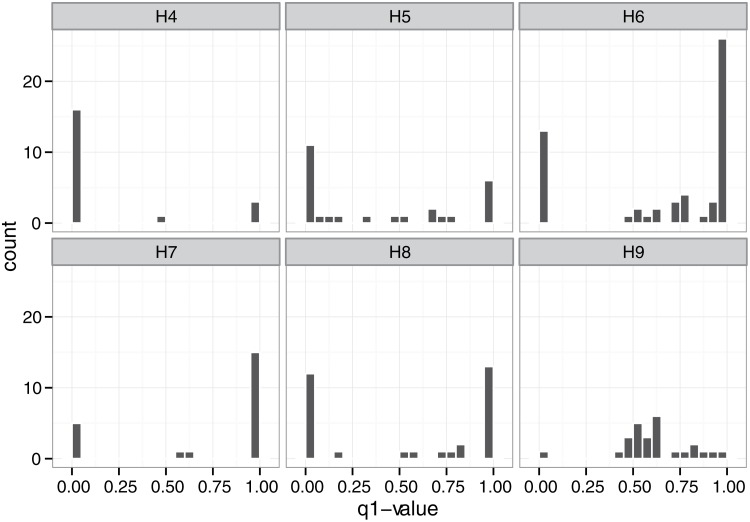
Frequency distribution of admixture coefficients (*q*_*1*_) for microsatellite genotypes of the individuals in each mixed population.

In the admixture analysis with STRUCTURE 2.2.3, Δ*K* values suggested that *K* = 2 is the optimal value of *K* (S1 Fig.). The two clusters corresponded well to the two morphological parental species, *L*. *japonicum* and *L*. *stellipilum* (Fig. 3a). Allopatric populations of *L*. *japonicum* and *L*. *stellipilum* were composed of purebred individuals (except one from population S1), which displayed a high assignment probability to their respective species groups, with mean values of 0.989 (range: 0.963–0.993) and 0.979 (range: 0.836–0.994), respectively. Individuals from sympatric populations of *L*. *japonicum* (H1 and H2) and *L*. *stellipilum* (H3) also showed high assignment probability with mean values of 0.980 (range: 0.899–0.995) and 0.990 (range: 0.980–0.995), respectively. All mixed populations composed of individuals displaying high assignment probabilities (*q*_*1*_ > 0.9 or *q*_*1*_ < 0.1) indicated parental species clusters and intermediate assignment probabilities (0.1 < *q*_*1*_ < 0.9) indicated hybrids. Fig. 4 shows the frequency distributions of the *q*_*1*_-value of the populations in the contact zone based on STRUCTURE. Population H9 was composed of numerous intermediate genotypes and showed a unimodal distribution of *q*_*1*_-values (dip test for unimodality: *D* = 0.0806, *P* = 0.175). In contrast, populations H4 and H7 were dominated by the parental genotype with few intermediates. Population H4 was dominated by the *L*. *stellipilum* genotype, while H7 was dominated by the *L*. *japonicum* genotype. Although few individuals of intermediate genotype occurred in these populations, the dip test failed to reject the null hypothesis that the distribution of admixture proportions was unimodal (S3 Table), which may have been due to an unbalanced local abundance of parental genotypes. Populations H5, H6, and H8 were mainly dominated by parental genotypes with a few intermediate genotypes, and therefore corresponded to a bimodal contact zone (dip test for unimodality: *P* < 0.0001 for populations H5, H6, and H8). Genetic constitutions of hybrids in H6 were closer to *L*. *japonicum*, while those of hybrids in H5 and H8 were relatively continuous, varying between *L*. *japonicum* and *L*. *stellipilum*.

### Performance of NEWHYBRIDS with simulated genotypes

An assignment test of the simulated data set revealed that most hybrids could be correctly identified as such by NEWHYBRIDS with a threshold value of 0.5 (S4 Table). All allopatric individuals (except one from population S1) had an assignment coefficient (*q*) higher than 0.90 in their respective cluster and were then used to obtain pure parental genotypes. The results regarding the performance of NEWHYBRIDS with the simulated genotypes are summarized in S4 Table. High performance levels were observed for simulated pure individuals in the genetic assignment with 98% for simulated pure individuals of *L*. *japonicum* and 96% for *L*. *stellipilum*.

For hybrid classes, the performance values were moderately lower in comparison with the pure parental genotype assignment. Eighty-five percent for simulated F_1_, 62% for F_2_, 82% for first generation backcrosses to *L*. *japonicum*, and 73% for first generation backcrosses to *L*. *stellipilum* individuals were assigned as such with a posterior probability over 0.5. The results demonstrated that the 10 microsatellite loci would be efficient for the assignment of individuals into parental classes, F_1_ hybrids, and first generation backcrosses to *L*. *japonicum*, but decreased the performance in assignment of the F_2_ class and first generation backcrosses to *L*. *stellipilum*.

### Genetic composition of the mixed populations

All allopatric *L*. *japonicum* and *L*. *stellipilum* individuals were assigned to their respective parental classes with NEWHYBRIDS except one individual in S1 (Fig. 3b). With the exception of one individual in H1 that may have been an introgressant, all individuals from sympatric *L*. *japonicum* and *L*. *stellipilum* populations were also assigned to parental classes (Fig. 3b, Table 3). The extent of hybridization varied among populations H4–H9 (Fig. 3b, Table 3). Population H9 was predominantly composed of intermediate hybrid classes (F_1_, F_2_, and F_n_) and first generation backcrosses to *L*. *japonicum*, with few parental classes. In contrast, populations H4 and H7 were composed mainly of parental classes with few intermediate hybrid classes. Populations H5, H6, and H8 were dominated by both parental classes and a few intermediate hybrid classes. In addition, in the two populations in which backcrossing occurred (populations H6 and H9), the direction of backcrossing was highly asymmetric to that of *L*. *japonicum*. The backcross classes in populations H6 and H9 consisted of more first generation backcrosses to *L*. *japonicum* and less first generation backcrosses to *L*. *stellipilum*.

**Table 3:** The number of *L*. *japonicum* (LJ), *L*. *stellipilum* (LS), F1, F2, advanced-generation hybrids (F_n_), first generation backcross to *L*. *japonicum* (BC1J), first generation backcross to *L*. *stellipilum* (BC1S), later generation backcross to *L*. *japonicum* genotypes (BCJ), later generation backcross to *L*. *stellipilum* (BCS) genotypes.

Genotype classes										
Population	LJ	LS	F1	F2	F_n_	BC1J	BC1S	BCJ	BCS	Total
H1	12	0	0	0	0	1	0	0	0	13
H2	15	0	0	0	0	0	0	0	0	15
H3	0	15	0	0	0	0	0	0	0	15
H4	3	16	1	0	0	0	0	0	0	20
H5	6	13	0	5	0	3	0	0	0	27
H6	26	13	1	5	0	10	1	0	0	56
H7	15	5	0	0	1	1	0	0	0	22
H8	13	12	0	4	1	2	0	0	0	32
H9	1	1	10	5	3	6	0	0	0	26
Total	91	75	12	19	5	23	1	0	0	226

## Discussion

### Genetic composition of hybrids

A previous study reported natural hybridization between *L*. *japonicum* and *L*. *stellipilum* based on cpDNA and ITS variations [64]. The assignments of the hybrid classes (Fig. 3b, Table 3) based on the microsatellite markers are consistent with the previous results of cpDNA and ITS variations overall [64]. However, only ribotype/cytotype combinations cannot elucidate F2 and first generation backcrosses. The present results successfully showed more detailed classification of hybrid classes.

The backcross BC1Js were much abundant than the BC1Ss (Fig. 3b, Table 3); BC1S was almost never detected. Such unidirectional backcrossing often resulted from crossing between F1 and the more abundant parent [39,86]. However this is not the case for the hybrid populations of *L*. *japonicum* and *L*. *stelipilum*. Another possible reason is that reproductive success tended to be greater in crossing between F1 and *L*. *japonicum* than crossing between F1 and *L*. *stelipilum* probably because of compatibility at stage of seed development.

### Frequency of hybridizaiton

Evidence from genetic distance-based clustering analysis, the frequency distribution of admixture proportion value (*q*), and the hybrid category assignment approaches indicated that the frequency and extent of hybridization varied considerably among populations in the contact zone between *L*. *japonicum* and *L*. *stellipilum*. In genetic distance-based clustering analysis, most individuals were clustered to *L*. *japonicum* or *L*. *stellipilum* in H4 and H7, while most individuals were located in the intermediate position in population H9. In populations H5, H6, and H8, individuals showed a continuous variation pattern covering both variation range of parental species and intermediate position. The frequency distributions of the *q*_*1*_-value based on STRUCTURE showed a unimodal distribution in population H9 but bimodal distributions in other populations (except population H4 and H7). Genotypic classes assigned by NEWHYBRIDS indicated that population H9 consisted largely of intermediate genotypes (F_1_, F_2_, and F_n_) and first generation backcrosses to *L*. *japonicum*, with few parental classes. In contrast, populations H4 and H7 were mainly composed of parental classes with few intermediate hybrid classes. Populations H5, H6, and H8 were dominated by both parental classes and a few intermediate hybrid classes. In addition, the divergent allele frequency between *L*. *japonicum* and *L*. *stellipilum* and relatively low frequency of hybridization within mixed populations may have given rise to significant heterozygote deficits at some loci in populations H4–H8 (Fig. 3, S2 Table). Significant heterozygote excess present at some loci in population H9 (S2 Table) may have been due to the high proportion of intermediate hybrids in H9.

Combined actions in the mechanisms of reproductive isolation may contribute to variation in modality of population structure and extent of hybridization, and may thus affect the evolutionary outcome of hybridization. Jiggins and Mallet [6] suggested that the bimodal contact zones are strongly associated with well-developed (but incomplete) pre-zygotic isolation, while unimodal contact zones show largely incomplete pre-zygotic isolation, including spatial isolation, temporal isolation, floral isolation, and gametic isolation [87]. Several scenarios could be responsible for the differences in between-site pre-zygotic isolation mechanisms. In *Pinus*, hybridization frequency is influenced by the density of vegetation between hybridizing species, which acts as a barrier to pollen dispersal [34]. In *Bruguiera* and *Narcissus*, hybridization frequency is influenced by the length of overlapping flowering period between two species in different localities [88–89]. In addition, floral morphology variation may lead to differences in pollinator preference between sites. In *Epimedium* species, species fidelity by nectar foraging bees dependent on nectar spur length; the difference in nectar spur length between populations can affect visit frequencies of nectar foraging bees and lead to variation in the strength of the ethological barrier [32]. Variation in pollinator preference can also be influenced by local microclimate. In *Ipomopsis* species, stronger pollinator fidelity in low-frequency hybrid sites than in high-frequency hybrid sites is caused by the local temperature. The warmer nighttime temperature in high-frequency hybrid sites allows the hawkmoths to forage nocturnally when white flowers of *I*. *tenuituba* are more visible. Thus, in the morning, the empty *I*. *tenuituba* flowers cause preference of hummingbirds for *Ipomopsis aggregata* [46]. In addition, differences in reproductive isolation can be caused by pollen performance, which is influenced by between-site abiotic factor differences. Abiotic factors can affect stylar chemical composition during maternal growth and pollen development [90–91]. Environment conditions, such as soil calcium (Ca) and water stress, were shown to lead to variations in pollen performance between two species of *Phlox* [47] and two species of *Quercus* [35]. In the present study, *L*. *japonicum* and *L*. *stellipilum* were distributed in close proximity in all populations, rendering no barrier to gene flow between the two species. Additionally, there is no difference in length of overlapping flowering period between two species in different localities. Furthermore, specific pollinator attraction mediated by floral traits is unlikely between *L*. *japonicum* and *L*. *stellipilum* because the floral traits are very similar between these two species. During field observations, we found a high flower visitation frequency mediated by their common pollinator *Bombus* sp., while visitation frequencies by other pollinators were very low. However, as we did not examine the local abiotic environmental factors in each population, such as, light condition, moisture, temperature, and so on, it is undeniable that one or a few of such factors may have influenced pollen performance within populations and the frequency of hybridization.

Fine-scale habitat differences could be responsible for the difference in between site post-zygote reproductive isolation. Between-population fine-scale habitat differences may result in the absence of habitats suitable for certain genotypes and lead to between-population differences in genetic structure and hybridization frequency. Although a lack of intermediate genotypes could be due to strong habitat differentiation, as suggested by Goulson and Jerrim [92], the absence of suitable habitat is sometimes inferred in some study systems, but not quantified. In *Rhododendron*, the F_1_-dominated hybrid zone between *R*. *caucasicum* and *R*. *ponticum* may be maintained by habitat-mediated superiority of F_1_s over all other genotype classes. In other examples, the same parent species can form multigeneration hybrids [14]. In another instance, recurrent floods may have prevented hybridization between *R*. *eriocarpum* and *R*. *indicum* in riverside compared to seaside areas [93]. In this study, the extent of hybridization varied among populations. Population H9 predominantly consisted of intermediate hybrid classes (F_1_, F_2_, and F_n_) and first generation backcrosses to *L*. *japonicum*, with few parental classes. In contrast, populations H4 and H7 were mainly composed of parental classes with few intermediate hybrid classes. Populations H5, H6, and H8 were dominated by both parental classes and a few intermediate hybrid classes. *Leucosceptrum japonicum* and *L*. *stellipilum* may adapt to the myriad of ecological factors in their specific habitats, although distinct difference between the habitats of *L*. *japonicum* and *L*. *stellipilum* were not found by visual inspection at our field sampling. Some ecological factors that cannot be recognized by visual inspection (such as nitrogen, metal ion concentration and pH of soil) may have contributed to the fitness of hybrids and parental species. Comparison of habitat variables by experimental approach between populations that exhibit different genotype distribution may provide insight into whether variation in environment-mediated selection will result in differences in the degree of reproductive isolation between *L*. *japonicum* and *L*. *stellipilum*.

## Conclusions

Climate oscillations during the Quaternary period are considered to affect plant species’ range distribution [94]. They may also have been associated with hybridization at the margin of the range of previously geographical isolated species [95–98]. One possible scenario to account for the formation of the contact zone between *L*. *japonicum* and *L*. *stellipilum* is that climate oscillations during the Quaternary period may have been associated with vicariance and secondary contact between the two species. During glaciation, *L*. *japonicum* and *L*. *stellipilum* may have retreated to separate refugia and undergone divergent evolution. During warm interglacial periods, both species probably expanded their distribution ranges and formed a contact zone which comprised by some small scale contact sites. The frequency and extent of hybridization varied considerably among these mixed populations. One likely explanation is that variation in exogenous (ecological) selection among populations might contribute to varying levels of strengths of pre-zygotic and/or post-zygotic reproductive isolation and lead to differences in frequency and extent of hybridization. The present study will facilitate future research exploring the evolution of reproductive isolation between *L*. *japonicum* and *L*. *stellipilum*.

## Supporting Information

S1 Table Pairwise estimates of genetic differentiation (F_ST_) between eight populations of *L*. *japonicum* and *L*. *stellipilum* base on 10 microsatellite markers. All pairwise *F*_ST_ estimates were significantly different from zero (*P* < 0.05).
(DOC)

S2 Table Inbreeding coefficient for all populations and ten microsatellite loci. (DOC)

S3 Table Results of dip test of admixture proportions (*q*_1_-value) calculated in STRUCTURE in populations H4 to H9. (DOC)

S4 Table Number of simulated individuals that were assigned respectively to the L. japonicum (LJ), L. stellipilum (LS), F1, F2, first generation backcrosses to L. japonicum (BC1J) and first generation backcrosses to L. stellipilum (BC1S) with NEWHYBRIDS. (DOC)

S1 Fig Bayesian inference of the most likely number of clusters in the STRUCTURE analysis. (a) Distribution of delta *K* for each *K* estimated from following Evanno et al. [74]. (b) Plot of mean likelihood logarithmic probability of the data using 10 replicates runs at each value of *K* (*K* = 1–10).
(DOC)
