# Supplementary material for: Variation in the Frequency and Extent of Hybridization between Leucosceptrum japonicum and L. stellipilum (Lamiaceae) in the Central Japanese Mainland
Source: PLoS One. 2015 Mar 4;10(3):e0116411. doi: 10.1371/journal.pone.0116411 (PMC4349587; doi:10.1371/journal.pone.0116411)
Supplement: S1 Table — All pairwise F ST estimates were significantly different from zero (P < 0.05). (DOC) [file pone.0116411.s001.doc]

**Table S1** Pairwise estimates of genetic differentiation (*F*_ST_) between eight populations of *L. japonicum* and *L. stellipilum* base on 10 microsatellite markers. All pairwise *F*_ST_ estimates were significantly different from zero (*P* < 0.05).

|  | J1 | J2 | S1 | S2 | S3 | H1 | H2 |
| --- | --- | --- | --- | --- | --- | --- | --- |
| J1 | - |  |  |  |  |  |  |
| J2 | 0.18 | - |  |  |  |  |  |
| S1 | 0.35 | 0.30 | - |  |  |  |  |
| S2 | 0.36 | 0.32 | 0.05 | - |  |  |  |
| S3 | 0.34 | 0.29 | 0.05 | 0.08 | - |  |  |
| H1 | 0.16 | 0.18 | 0.31 | 0.32 | 0.30 | - |  |
| H2 | 0.25 | 0.22 | 0.29 | 0.32 | 0.31 | 0.22 | - |
| H3 | 0.37 | 0.34 | 0.11 | 0.10 | 0.14 | 0.35 | 0.36 |
