# Supplementary material for: Variation in the Frequency and Extent of Hybridization between Leucosceptrum japonicum and L. stellipilum (Lamiaceae) in the Central Japanese Mainland
Source: PLoS One. 2015 Mar 4;10(3):e0116411. doi: 10.1371/journal.pone.0116411 (PMC4349587; doi:10.1371/journal.pone.0116411)
Supplement: S2 Table — (DOC) [file pone.0116411.s002.doc]

**Table S2** Inbreeding coefficient for all populations and ten microsatellite loci.

|  |  |  |  |  | Locus |  |  |  |  |  |
| --- | --- | --- | --- | --- | --- | --- | --- | --- | --- | --- |
| Populations | Leu1 | Leu2 | Leu3 | Leu4 | Leu5 | Leu6 | Leu7 | Leu8 | Leu9 | Leu10 |
| J1 | 0.091 | 0.008 | -0.041 | - | 0.177 | 0.108 | 0.020 | 0.258 | 0.196 | 0.033 |
| J2 | -0.052 | 0.042 | 0.113 | 0.097 | 0.153 | -0.077 | 0.041 | - | 0.003 | 0.174 |
| S1 | 0.120 | 0.107 | 0.122 | 0.014 | 0.215 | -0.088 | - | - | 0.284 | 0.121 |
| S2 | -0.030 | 0.087 | 0.188 | -0.127 | 0.186 | 0.750 | - | - | 0.083 | 0.051 |
| S3 | 0.070 | 0.105 | 0.015 | 0.043 | -0.055 | 0.069 | - | - | 0.195 | 0.161 |
| H1 | -0.263 | 0.197 | 0.029 | 0.467 | 0.115 | -0.023 | 0.127 | 0.733*** | 0.107 | 0.118 |
| H2 | -0.037 | 0.119 | 0.202 | 0.312* | -0.134 | -0.166 | 0.634* | 0.354 | 0.217 | 0.243 |
| H3 | 0.116 | 0.155 | 0.269 | 0.266 | 0.170 | -0.188 | - | - | 0.049 | -0.034** |
| H4 | 0.042 | 0.174** | 0.436*** | 0.456*** | 0.149 | 0.097*** | 0.835** | 0.791*** | 0.101* | 0.240*** |
| H5 | 0.387*** | 0.021 | 0.074*** | 0.606*** | 0.112 | 0.748*** | 0.281*** | 0.655*** | 0.639*** | 0.583*** |
| H6 | 0.082*** | 0.298*** | 0.169*** | 0.358*** | 0.278*** | 0.390*** | 0.304** | 0.639*** | 0.319*** | 0.422*** |
| H7 | 0.133*** | 0.603*** | -0.104*** | 0.440*** | -0.042 | 0.747*** | 0.388** | 0.313*** | -0.099*** | -0.126*** |
| H8 | 0.431*** | -0.134 | 0.143*** | 0.480** | 0.555*** | 0.139** | 0.766*** | 0.635*** | 0.216*** | 0.279*** |
| H9 | -0.288*** | 0.045*** | 0.014*** | -0.096 | -0.585*** | 0.231*** | -0.34*** | -0.473*** | -0.140*** | -0.174 |
| **P* < 0.05, ***P* < 0.01, ****P* < 0.001 | | |  |  |  |  |  |  |  |  |
