# Supplementary material for: Variation in the Frequency and Extent of Hybridization between Leucosceptrum japonicum and L. stellipilum (Lamiaceae) in the Central Japanese Mainland
Source: PLoS One. 2015 Mar 4;10(3):e0116411. doi: 10.1371/journal.pone.0116411 (PMC4349587; doi:10.1371/journal.pone.0116411)
Supplement: S3 Table — (DOC) [file pone.0116411.s003.doc]

**Table S3** Results of dip test of admixture proportions (*q_1_*-value) calculated in STRUCTURE in populations H4 to H9.

| Site | *D* | *P* value |
| --- | --- | --- |
| H4 | 0.0750 | 0.4615 |
| H5 | 0.1108 | < 0.0001 |
| H6 | 0.1117 | < 0.0001 |
| H7 | 0.1118 | 0.0163 |
| H8 | 0.1839 | < 0.0001 |
| H9 | 0.0806 | 0.1749 |
