# Supplementary material for: Variation in the Frequency and Extent of Hybridization between Leucosceptrum japonicum and L. stellipilum (Lamiaceae) in the Central Japanese Mainland
Source: PLoS One. 2015 Mar 4;10(3):e0116411. doi: 10.1371/journal.pone.0116411 (PMC4349587; doi:10.1371/journal.pone.0116411)
Supplement: S4 Table — (DOC) [file pone.0116411.s004.doc]

**Table S4** Number of simulated individuals that were assigned respectively to the *L. japonicum* (LJ), *L. stellipilum* (LS), F1, F2, first generation backcrosses to *L. japonicum* (BC1J) and first generation backcrosses to *L. stellipilum* (BC1S) with NEWHYBRIDS.

| Simulated/assigned | P1 | P2 | F1 | F2 | BC-P1 | BC-P2 | unassigned | Total |
| --- | --- | --- | --- | --- | --- | --- | --- | --- |
| LJ | 50 | 0 | 0 | 0 | 0 | 0 | 0 | 50 |
| LS | 0 | 49 | 0 | 0 | 0 | 1 | 0 | 50 |
| F1 | 0 | 0 | 47 | 1 | 0 | 0 | 2 | 50 |
| F2 | 0 | 0 | 2 | 33 | 7 | 8 | 0 | 50 |
| BLJ | 1 | 0 | 2 | 0 | 47 | 0 | 0 | 50 |
| BLS | 0 | 1 | 1 | 1 | 0 | 44 | 3 | 50 |
| Total | 51 | 50 | 52 | 35 | 54 | 53 | 5 |  |
| Efficiency | 0.98 | 0.98 | 0.90 | 0.94 | 0.87 | 0.83 |  |  |
| Accuracy | 1.00 | 0.98 | 0.94 | 0.66 | 0.94 | 0.88 |  |  |
| Performance | 0.98 | 0.96 | 0.85 | 0.62 | 0.82 | 0.73 |  |  |
