# Supplementary material for: Variation in the Frequency and Extent of Hybridization between Leucosceptrum japonicum and L. stellipilum (Lamiaceae) in the Central Japanese Mainland
Source: PLoS One. 2015 Mar 4;10(3):e0116411. doi: 10.1371/journal.pone.0116411 (PMC4349587; doi:10.1371/journal.pone.0116411)

**Figure S1** Bayesian inference of the most likely number of clusters in the STRUCTURE analysis. (a) Distribution of delta *K* for each *K* estimated from following Evanno et al. [74]. (b) Plot of mean likelihood logarithmic probability of the data using 10 replicates runs at each value of *K* (*K*= 1-10).


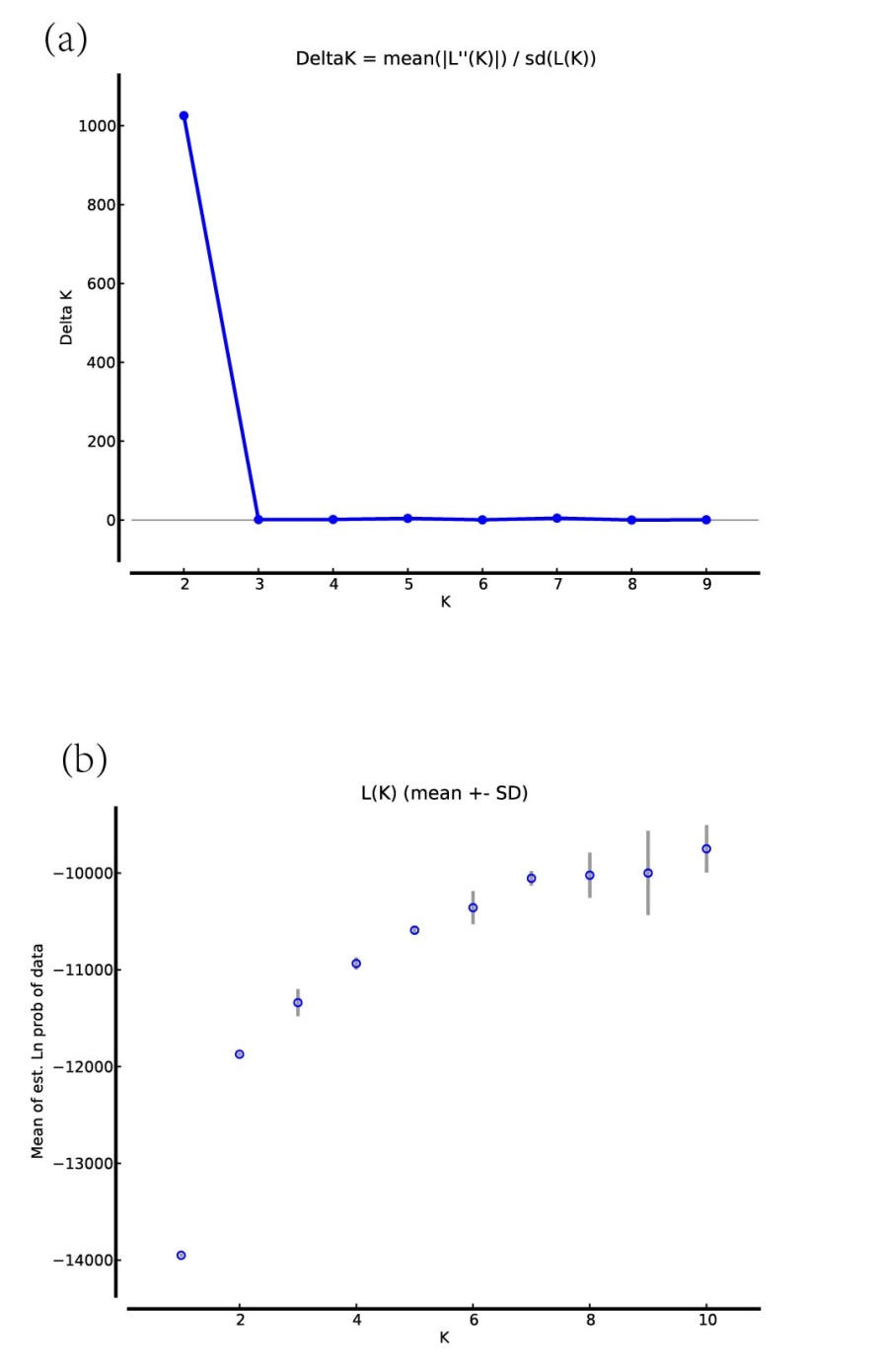

Supplement: S1 Fig — (a) Distribution of delta K for each K estimated from following Evanno et al. [74]. (b) Plot of mean likelihood logarithmic probability of the data using 10 replicates runs at each value of K (K = 1–10). (DOC) [file pone.0116411.s005.doc]
